# Profiles of centenarians’ functioning: linking functional and cognitive capacity with depressive symptoms

**DOI:** 10.1186/s12877-024-05036-8

**Published:** 2024-05-23

**Authors:** Kim Uittenhove, Charikleia Lampraki, Carla Gomes da Rocha, Christoph Rott, Armin von Gunten, Daniela S. Jopp

**Affiliations:** 1 Institute of Psychology, University of Lausanne & Swiss Centre of Expertise in Life Course Research, Bâtiment Géopolis, Lausanne, CH-1015 Switzerland; 2 Faculty of Psychology and Educational Sciences, University of Geneva, Geneva, Switzerland; 3 Service of Old Age Psychiatry, Lausanne University Hospital and University of Lausanne, Lausanne, Switzerland; 4 School of Health Sciences, HES-SO Valais-Wallis, Sion, Switzerland; 5 Institute of Biomedical Sciences Abel Salazar, University of Porto, Porto, Portugal; 6 Institute of Gerontology, Heidelberg University, Heidelberg, Germany

**Keywords:** Gerontology, Centenarians, Depressive symptoms, Functional capacity, Cognitive capacity

## Abstract

**Background:**

Despite most centenarians facing age-related declines in functional and cognitive capacities, the severity of these declines varies among individuals, as does the maintenance of good mental health (e.g., depressive symptoms) despite these declines. This study aims to examine this heterogeneity in centenarians from the Second Heidelberg Centenarian Study, which collected data from 112 centenarians living in Germany. In our study, we focus on a subsample of 73 centenarians who provided self-reports for our measures of interest (*M* age = 100.4, *SD* age = 0.55).

**Methods:**

We examined correlations between functional capacity (i.e., PADL, IADL), cognitive capacity (i.e., MMSE), and depressive symptoms (i.e., GDS), and the existence of different profiles using hierarchical clustering.

**Results:**

Higher functional capacity was related to higher cognitive capacity and to fewer depressive symptoms. Yet, higher cognitive capacity was associated with *more* depressive symptoms. Hierarchical clustering analysis elucidated this contradiction by identifying three profiles: *low-capacity* individuals (i.e., 24 individuals had low functional and cognitive capacities, with low depressive symptoms), *high-capacity* individuals (i.e., 33 individuals with high functional and cognitive capacities, with low depressive symptoms), and *low-functional-high-cognitive-capacity* individuals (i.e., 16 individuals showed low functional but high cognitive capacity, with high depressive symptoms). Our post-hoc analyses highlighted arthritis and pain as risk factors for functional dependence and depression.

**Conclusions:**

Our findings emphasize the importance of identifying centenarian subgroups with specific resource- and risk profiles to better address their needs, and of treating pain to improve functional capacity and mental health in centenarians.

**Supplementary Information:**

The online version contains supplementary material available at 10.1186/s12877-024-05036-8.

## Introduction

### Cognitive and functional capacity in centenarians

Centenarians, often considered a paragon of successful aging due to their remarkable longevity, exhibit nonetheless considerable variation in their functional and cognitive abilities [1–4]. These abilities not only impact survival [5, 6] but also influence aspects critical to quality of life, including autonomous living [7] and well-being [8–10]. Numerous studies have observed a moderately positive association between functional and cognitive capacity in centenarians [11] see also [12, 13]). Preserved cognitive function is considered essential for functional independence in centenarians [11, 14], whereas cognitive impairment represents a risk factor for functional dependence [15]. However, there remains significant variability as centenarians at different levels of cognitive ability often exhibit a broad spectrum of functional dependence [12, 14].

### The impact of Functional and Cognitive decline on Mental Health

Only around 20% of centenarians exhibit sufficient functional and cognitive capacity to maintain sufficient independence to live alone without any informal or professional assistance [7, 16, 17]. In fact, a sizable proportion of centenarians encounter moderate to severe cognitive and functional impairments, with a median prevalence of dementia around 50% [3, 4, 18]. Furthermore, between 40 and 60% of individuals aged 100 or older experience limitations in performing basic Activities of Daily Living (ADL), whereas over 90% face challenges with more complex Instrumental Activities of Daily Living (IADL) as reported in several studies [7, 11, 19, 20].

Despite experiencing age-related declines in functional and cognitive capacities, many centenarians continue to exhibit good mental health and well-being, showcasing remarkable psychological resilience [9, 20–23]. Psychological resilience in very old adults is influenced by how they adapt to age-related losses in physical and cognitive capacities. This resilience is crucial for maintaining mental well-being despite these declines. For example, Rothermund and Brandstädter [24] highlight the importance of emotion and cognition regulation strategies to help buffer the negative impacts of performance decline in very old adults, leading to better well-being outcomes. Other studies also show that older adults, including those with disabilities or deteriorating health, benefit from adjusting their goals to maintain well-being [25–27]. This might be particularly important for centenarians. Despite accumulation of age-related losses [28], they seem to experience less impact on their mental health from functional limitations and disabilities compared to younger older adults (aged 60–90 years) [22, 23]. Despite this adaptation, many studies still find links between the decline in functional and cognitive abilities and decreased mental health or well-being in centenarians. Higher functional and cognitive capacities are often associated with greater subjective well-being [9, 10, 21], less depressive symptoms [9, 29], and more positive affect [8, 30].

The process of dealing with age-related losses is likely not uniform among centenarians, which might account for the inconsistent findings in studies examining the relationship between functional and cognitive capacities and overall well-being [31]. The relationship between these objective capacities and subjective mental health indicators, such as depressive symptoms, may differ among different subgroups of centenarians. Therefore, considering subgroups of mental health in conjunction with functional and cognitive capacities may offer a more comprehensive understanding of the heterogeneity in this population.

### Centenarians’ capacity profiles

Examining only broad sample associations can obscure individual differences in centenarians’ capacity profiles, which may comprise different levels of functional and cognitive capacities and mental health. Some studies have identified distinct capacity profiles among centenarians. For example, Alvarez et al., (2021) [32] classified centenarians into “Robust,” “Frail,” and “Intermediate” profiles, based on health (i.e., physical: Chair Stand Test; and functional: Katz’s Disability Index) and cognitive (i.e., MMSE) abilities. Other studies have additionally distinguished groups that exhibit elevated depressive symptoms in conjunction with reduced functional and cognitive capacities [33–35].

A more person-centered approach delineating capacity profiles could better capture the unique experiences of centenarians compared to focusing on identifying broad relationships between various indicators of functioning in samples of centenarians. Nonetheless, given that such findings are greatly dependent on the specific sample studied, it is important to extend previous findings to other samples. As such, our study aims to further the understanding of centenarians’ capacity profiles by examining functional capacity, cognitive capacity, and mental health in a representative, population-based sample.

### Current study

In this study, we examined a sample of centenarians from the Second Heidelberg Centenarian Study (HD100-II; [28, 36]) to investigate centenarians’ capacity profiles. We first analyzed associations between functional capacity, cognitive capacity and depressive symptoms within the entire sample and then identified subgroups with specific profiles reflecting combinations of these variables. Drawing from previous research on centenarian profiles or classes, we anticipated identifying profiles representing overall low or high functioning across these dimensions [32–35]. However, the mental health outcomes associated with particular levels of functional and cognitive capacity might not be uniform among all centenarians. For example, profiles could differ in the sustaining of good mental health (i.e., minimal depressive symptoms) despite diminished functional and cognitive capacity. Finally, we explored health domains linked to the various profiles by conducting a post-hoc analysis with health-related variables, such as the presence of sensory and mobility diseases, arthritis, and pain. These factors are known to affect functional and cognitive abilities and depressive symptoms, as evidenced in previous studies [37–40].

## Materials and methods

### Study sample

The HD100-II Study comprises 112 centenarians sampled from the population living in a 60-km radius around the city of Heidelberg, Germany. The Institutional Review Board of the Heidelberg University approved the study procedures and informed consent was obtained for all participants. Study participation involved two in-person interviews conducted at the participant’s residence. As there was no exclusion criterion, the HD100-II Study contained centenarians with a wider range of cognitive functioning. Of the total sample, 18 centenarians were entirely unable to respond to any interview questions and 21 others did not answer the questions assessing depressive symptoms due to limited cognitive capacity, poor hearing or health, or premature interview termination initiated either by the participant or because the interview was infeasible. Given that our study required these reports, we excluded those cases and focused on a sample of 73 centenarians who provided self-reports for our measures of interest, a common necessity in research of this nature [33]. Table 1 presents demographic characteristics for the HD100-II sample as well as the subsample of the current study, as well as levels of cognitive capacity, functional capacity, and depressive symptoms.

We conducted a sample selectivity analysis in accordance with Jopp and Rott [23]. These analyses aimed to compare the individuals who were part of the present sample to the full HD100-II sample. Selectivity between the HD100-II sample and the included sample was calculated as follows: [*M*_included_ – *M*_HD100−II_]/*SD*_HD100−II_. Selectivity effects are shown, expressed in standard deviation units (*d*), in the last column of Table 1. It is of note that we did not conduct sample selectivity analysis for depressive symptoms, given that we did not have these data for the excluded sample. Absent or small selectivity effects were observed for age, gender, education years, and residence, with effects ranging from − 0.21 to 0.22. Medium selectivity effects were observed for functional capacity (PADL and IADL), with effects of 0.39 for both indicators. Large selectivity effects (0.60) were observed for cognitive capacity. Thus, the subsample included in the current study presented higher levels of functional and cognitive capacity than the full sample and therefore results may not be generalizable to centenarians with very low levels of cognitive and functional capacity.

### Measures

#### Functional capacity

We measured functional health with the Older Americans Resources and Services (OARS) Multidimensional Functional Assessment Questionnaire [41], in which participants indicated how much difficulty they have performing seven personal activities of daily living (PADLs) and seven instrumental activities of daily living (IADLs) using a 3-point rating scale (0 = can’t do without help, to 2 = no difficulty; 0–14 each). We calculated sum scores for PADL and IADL capacity, with higher numbers indicating higher functional capacity. Both centenarians and proxies provided answers to these questions, and when applicable, we gave priority to proxy reports (61.1% of cases). This decision was based on prior research indicating that centenarians tend to overestimate their ADL capacities. Additionally, it has been observed that self-ratings are heavily influenced by the individuals’ current mood levels [42].

#### Cognitive capacity

We evaluated cognitive capacity by a short version of the Mini-Mental State Examination (MMSE; [43]). The use of a short MMSE reduces bias that stems from sensory and motor impairments while maintaining good psychometric properties and accuracy, making it a useful tool in centenarian studies [3, 44]. This short version has a maximum score of 21 and includes Orientation (10 points), Registration (3 points), Attention/Calculation (5 points), and Recall (3 points). Higher numbers indicate higher cognitive capacity.

### Depressive symptoms

We assessed depressive symptoms with a 10-item version of the Geriatric Depression Scale (GDS; [45]). An example item is “Do you feel your situation is hopeless?”. Participants responded “yes” (= 1) or “no” (= 0). On average, participants had 4.9% missing values. We replaced these missing values using predictive mean matching performed with the ‘mice’ package in R [46]. We subsequently calculated the sum of the different items for each centenarian, with higher scores indicating more depressive symptoms. In parallel to a dimensional approach, we explored depression severity. Our study did not allow us to establish a clinical diagnosis of depression or depression subtypes. However, an indication of depression severity was obtained by extrapolating cut-off criteria established for the GDS-30, where scores from 0 to 9 suggest that the individual is normal or nondepressed, scores between 10 and 19 indicate mild to moderate depression, and scores ranging from 20 to 30 indicate severe depression. For the GDS-10 used in our study, we proportionally adjusted the cut-off points, where scores from 0 to 2 indicate no depression, scores from 3 to 6 reflect mild to moderate depression, and scores from 7 to 10 suggest severe depression.

### Health variables

In our post-hoc analyses, we examined different health issues that could interfere with functional and cognitive capacities as well as depressive symptoms, according to prior work. These health variables included the presence of sensory issues (i.e., present = 1, absent = 0, if one or more of the following was present: hearing impairment, vision impairment, vision disease such as glaucoma, cataract, macular degeneration), the presence of a mobility issue (i.e., present = 1, absent = 0, if one or more of the following was present: difficulty with walking, mobility, balance, falls), and the presence of arthritis, as leading pain-provoking condition (i.e., diagnosis present = 1, absent = 0. In addition, we included pain strength (i.e., measured on an 11-point scale going from no pain = 0 to unbearable = 10).

### Analysis strategy

The first part of our analysis examined correlations between functional capacity, cognitive capacity, and depressive symptoms, as well as combinations of these variables using hierarchical clustering. As a first step, this entailed investigating individual levels of functional capacity, cognitive capacity, and depressive symptoms, and examining associations through Spearman correlation analyses with SPSS. As a second step, we identified capacity profiles, combining functional capacity, cognitive capacity, and depressive symptoms, by performing hierarchical cluster analysis using the ‘HCPC’ function from the ‘FactoMineR’ package in R [47]. Details on this method are outlined in Supplemental File 1. Our study aimed to uncover new insights into centenarian capacity profiles and the exploratory nature of hierarchical clustering aligned with these objectives, by identifying natural groupings in data without a priori expectations about the underlying structure of the data. Given the exploratory nature of our study objective, and our use of three well-defined variables that capture key aspects that distinguish how centenarians function, our sample size of 73 participants was suitable for this type of analysis. Of key importance is whether hierarchical clustering analysis yielded interpretable and stable clusters in our study. Supplemental File 1 contains Ward’s dendrogram, which visualizes the data grouping process and the proximity between individual observations, as well as a silhouette analysis and bootstrapping, which indicate a fair clustering structure that is robust across variations in sample composition. To characterize the differences in capacity levels of the resulting clusters, we compared functional capacity, cognitive capacity and depressive symptoms through ANOVAs with post-hoc tests adjusted for multiple comparisons (Bonferroni). In addition, we used the non-parametric Kruskal-Wallis test to confirm our findings.

The second part of our analysis was performed post-hoc with the objective of deepening our understanding of the different capacity profiles. As a first step, we conducted a post-hoc analysis that examined whether the profiles differed in sociodemographic (i.e., age, gender, living situation, education years) and health variables (i.e., sensory issues, mobility issues, arthritis, pain strength). For analyzing the difference between profiles for dichotomous variables, we used the Fisher-Freeman-Halton Exact Test in SPSS. This test is appropriate for contingency tables that are larger than 2 × 2 and for small sample sizes [48]. It offers a higher degree of rigor and precision in cases where the assumptions of the chi-squared test may not hold, by calculating the exact p-value for the observed data. For analyzing the difference between capacity profiles for continuous variables, we conducted ANOVAs with post-hoc tests adjusted for multiple comparisons (Bonferroni) as well as a Kruskal-Wallis test to confirm the findings. As a second step, we conducted Spearman correlations between health variables and individual levels of functional capacity, cognitive capacity, and depressive symptoms, as well as partial correlations controlling for health variables. Statistical significance was set at *p* < .05, with a 95% confidence interval.

## Results

### Descriptive analysis and correlations

Mean levels and standard deviations for functional capacity, cognitive capacity, and depressive symptoms can be found in Table 1. Concerning functional capacity, the present sample displayed an average score of 9.89 for PADL (range 3–14), and a lower average score of 6.26 for IADL (range 0–14). Concerning cognitive capacity, our subsample obtained an average score of 16.60 (4–21) for the MMSE. Concerning depressive symptoms, our subsample reported an average of 2.19 (0–9) on the GDS. The theoretical range for each variable can be found in Table 1.

To reflect functional capacity, we summed ADL and IADL scores. Spearman correlation analysis revealed that higher functional capacity was associated with higher cognitive capacity (*r* = .37, *p* < .01). Higher functional capacity was also associated with lower depressive symptoms (*r* = − .27, *p* < .05). It is of note that despite being associated to higher functional capacity, higher cognitive capacity was associated to more depressive symptoms (*r* = .27, *p* < .05).

**Table 1:** Characteristics of the full HD100-II sample as well as of the sample of participants included in this study

	HD100-II (*N* = 112)		Included (*n* = 73)		Selectivity
	*M/%*	*SD*	*M/%*	*SD*	*d*
Demographic Variables					
Age	100.5	0.47	100.4	0.55	-0.21
Gender: female	89.3	30.9	84.9	35.8	-0.14
Gender: male	10.7		15.1		
Education years	9.39	2.78	9.64	3.14	0.09
Residence: community	60.7	48.8	71.2	45.3	0.22
Residence: institution	39.3		28.8		
Functional Capacity					
PADL (0–14)	8.36	3.91	9.89	2.94	0.39
IADL (0–14)	4.72	3.91	6.26	3.67	0.39
Cognitive Capacity					
MMSE (0–21)	12.22^1^	7.25	16.60	3.62	0.60
Depressive Symptoms					
GDS (0–10)	2.10	2.29	2.10	2.29	

Note.^1^18 participants had severe cognitive impairment, as reflected in their incapacity to respond to MMSE questions and were attributed a 0 score

There were no missing values for the included sample. Missing values for the excluded sample were as follows: education years: *n* = 3; MMSE: *n* = 1; IADL: *n* = 1; GDS: *n* = 39.

### Capacity Profile Analysis

**Fig. 1:**
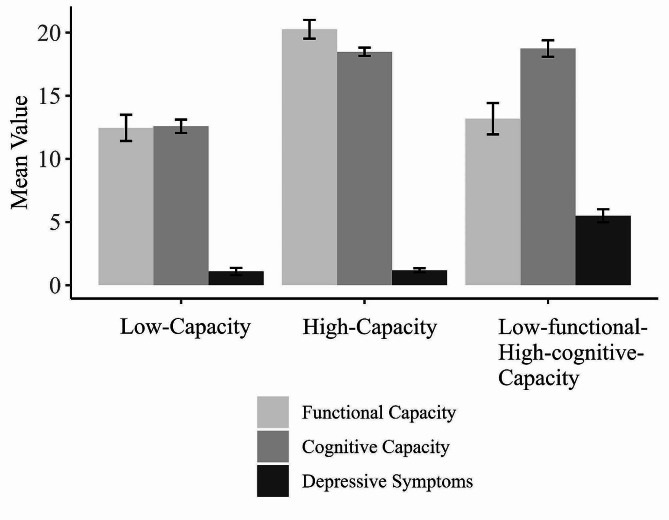
Profiles of functional capacity, cognitive capacity, and depressive symptoms

Next, we examined the existence of capacity profiles, combining functional capacity, cognitive capacity, and depressive symptoms. Ward’s dendrogram and the analysis of inter-cluster inertia gain indicated that the most optimal solution consisted of three clusters. Our subsequent examination confirmed that this clustering demonstrated fairly good cohesion and separation and was robust against sample variability (see Supplemental File 1). The levels of functional capacity, cognitive capacity, and depressive symptoms for the three clusters are reported in Table 2. We describe each cluster in terms of how their characteristics compare to the sample. The first cluster consisted of a *low-capacity profile* with individuals who displayed comparatively low functional and cognitive capacity, as well as low depressive symptoms. A second cluster consisted of a *high-capacity profile* with individuals who displayed relatively high functional and cognitive capacity, as well as low depressive symptoms. A third cluster revealed a *low-functional-high-cognitive-capacity profile*, with individuals who, compared to the sample, exhibited low functional yet high cognitive capacity, as well as high depressive symptoms. Figure 1 depicts the different profiles.

#### Note

The possible range of scores was 0–14 for functional capacity, 0–21 for cognitive capacity, and 0–10 for depressive symptoms.

Regarding the characterization of the different profiles based on their respective capacity levels, ANOVAs confirmed significant differences for functional capacity (*F*(2,70) = 23.27, *p* < .001, *η2* = 0.40), for cognitive capacity (*F*(2,70) = 55.15, *p* < .001, *η2* = 0.61), and for depressive symptoms (*F*(2,70) = 59.94, *p* < .001, *η2* = 0.63). Post-hoc tests adjusted for multiple comparisons (Bonferroni) indicated that concerning functional capacity, low-capacity and low-functional-high-cognitive-capacity individuals did not significantly differ from each other, but high-capacity individuals scored significantly higher than these other groups (*p* < .001). Concerning cognitive capacity, high-capacity and low-functional-high-cognitive-capacity individuals did not significantly differ from each other, but low-capacity individuals scored significantly lower than these other groups (*p* < .001). Finally, concerning depressive symptoms, low-capacity and high-capacity individuals did not significantly differ from each other, but low-functional-high-cognitive-capacity individuals scored significantly higher than these other groups (*p* < .001). These findings were replicated with the non-parametric Kruskal-Wallis test. Concerning the potential severity of depression, the low- and high-capacity groups contained 4 (16.7%) and 2 (8.3%) individuals, respectively, who met the cut-off for mild to moderate depression, whereas the low-functional-high-cognitive-capacity group contained 11 (68.8%) individuals who met the cut-off for mild to moderate depression, as well as 4 individuals (25%) who met the cut-off for severe depression, and only one individual who did not meet the cut-off for depression. Therefore, the vast majority (93.9%) of low-functional-high-cognitive-capacity individuals met the cut-off for mild and even severe depression.

**Table 2:** Characteristics of the profiles identified by the hierarchical cluster analysis. Mean levels of functional capacity, cognitive capacity, and depressive symptoms

	Low-capacity group (*n* = 24)		High-capacity group (*n* = 33)		Low-functional-high-cognitive-capacity group (*n* = 16)		F
	M/*N*	SD/%	M/*N*	SD/%	M/*N*	SD/%	
Functional capacity (ADL + IADL)	12.46^b^	5.08	20.27^a^	4.28	13.19^b^	4.94	23.27
Cognitive Capacity (MMSE)	12.58^b^	2.59	18.48^a^	1.86	18.75^a^	2.62	55.15
Depressive Symptoms (GDS)	1.08^b^	1.41	1.18^b^	0.95	5.50^a^	2.07	59.94
No Depression (0–2)	20	83.3%	31	91.7%	1	6.1%	
Mild to Moderate Depression (3–6)	4	16.7%	2	8.3%	11	68.8%	
Severe Depression (7–10)	0	0%	0	0%	4	25%	

Note. The low-capacity group consisted of individuals with low functional and cognitive capacity, as well as low depressive symptoms. The high-capacity group consisted of individuals with high functional and cognitive capacity, as well as low depressive symptoms. The low-functional-high-cognitive-capacity group consisted of individuals with low functional yet high cognitive capacity, as well as high depressive symptoms. To indicate significant differences among groups, we used the indicator ^a^ for values that were significantly higher than values with the indicator ^b^ (*p* < .05)

### Sociodemographic and health variables Associated with the capacity profiles

We compared sociodemographic characteristics and health issues between capacity profiles. Table 3 contains age, gender, living situation, and years of education, as well as prevalence of sensory issues, mobility issues, arthritis and reported pain strength, for each profile. Fisher-Freeman-Halton exact tests did not reveal significant differences between the profiles for gender (*p* = .45) or living situation (*p* = .89), and ANOVAs showed no significant differences for age (*F*(2,70) = 0.11, *p* = .89) or for education years (*F*(2,70) = 1.99, *p* = .14). Fisher-Freeman-Halton exact tests did not reveal significant differences between the profiles for the prevalence of sensory issues (*p* = .15), but there was a tendency towards a significant difference for the prevalence of mobility issues (*p* = .06), with the low-functional-high-cognitive-capacity group showing a *lower* prevalence of mobility issues. Concerning pain conditions, we found significant difference for the prevalence of arthritis (*p* < .05), with the low-functional-high-cognitive-capacity profile showing a higher prevalence than the other groups. An ANOVA also showed a significant difference for pain strength (*F*(2,69) = 9.03, *p* < .001, *η2* = 0.21). Post-hoc tests adjusted for multiple comparisons (Bonferroni) indicated that the low-functional-high-cognitive-capacity group experienced significantly stronger pain than the other two groups (*p*s < 0.001), whereas these other two groups did not differ from each other. When applicable (i.e., age, years of education, pain strength), the non-parametric Kruskal-Wallis test confirmed the pattern of findings.

Arthritis and pain strength might contribute to reduced functional capacity and increased depressive symptoms. Therefore, we turned back to the full sample (*n* = 73) for a follow-up analysis including correlations and partial correlations among the key constructs of interest. Spearman correlations indicated that the presence of arthritis correlated with lower functional capacity (*r* = –.27, *p* < .05) and more depressive symptoms (*r* = .29, *p* < .05), but not with cognitive capacity (*r* = .16, *p* = .19). Additionally, more intense pain was linked to more depressive symptoms (*r* = .48, *p* < .001) and showed a near-significant positive relationship with cognitive capacity (*r* = .22, *p* = .07), while no significant association was found with functional capacity (*r* = –.10, *p* = .41). When controlling for arthritis and pain strength to test partial correlations, we found a strong correlation between functional and cognitive capacity (*r* = .51, *p* < .001), while correlations between depressive symptoms and cognitive capacity (*r* = .09, *p* = .46) and between depressive symptoms and functional capacity (*r* = –.17, *p* = .15) were not significant anymore. This suggests that the previously observed association between depressive symptoms and both cognitive and functional abilities was primarily due to pain-related issues.

**Table 3:** Health variables according to profile

	Low-capacity group (*n* = 24)		High-capacity group (*n* = 33)		Low-functional-high-cognitive-capacity group (*n* = 16)		
	*M/%*	*SD*	*M/%*	*SD*	*M/%*	*SD*	*p*
Age	100.45	0.51	100.45	0.67	100.37	0.32	0.89
Gender: female	91.7		78.8		87.5		0.45
Residence: community	75.0		69.7		68.8		0.89
Education years	9.29	2.63	10.39	3.94	8.63	0.96	0.14
Sensory issues	91.7		100.0		100.0		0.15
Mobility issues	87.5		84.8		56.3		0.06
Arthritis	41.7		51.5		87.5		< 0.05
Pain strength^1^	2.48	2.45	2.67	2.12	5.38	2.53	< 0.001

Note:^1^1 missing value for pain strength

## Discussion

This study identified correlations among functional capacity, cognitive capacity, and depressive symptoms in centenarians. In addition, it highlighted distinct centenarian profiles based on these capacities and pinpointed specific factors linked to combinations of functional and cognitive capacities and depressive symptoms. Notably, pain and arthritis emerged as significant associated characteristics. We delve into each of these findings in greater detail.

### Functional capacity, cognitive capacity, and depressive symptoms

According to existing theories and prior research, functional capacity depends on cognitive capacity, and declining functional and cognitive capacity are linked with an increase in depressive symptoms. Examining the correlations between functional capacity, cognitive capacity, and depressive symptoms, we found that centenarians’ higher functional capacity moderately correlated with increased cognitive capacity. This was in line with previous studies that have observed a similar moderately positive association between functional and cognitive capacity in centenarians [11–13]). Additionally, our findings were consistent with the literature in revealing a relationship between greater functional capacity and fewer depressive symptoms [9, 33]. However, an unexpected finding was the connection between *higher* cognitive capacity and *more* depressive symptoms. Looking at the profiles in more detail allowed us to better understand this apparent inconsistency.

### Different centenarian profiles

Our cluster analysis elucidated the pattern of correlations by identifying distinct patterns of functional capacity, cognitive capacity, and depressive symptoms in centenarians. Our analysis identified a low-capacity profile characterized by low functional and cognitive capacity, as well as a high-capacity profile with high functional and cognitive capacity, in line with prior research [32–35]. However, in our study, the low-capacity profile did not simultaneously have high depressive symptoms. Instead, individuals in the low-capacity group exhibited similarly low levels of depressive symptoms as those with a high-capacity profile. In addition, our study also uniquely revealed a low-functional-high-cognitive-capacity group not identified in earlier research. These individuals possessed high cognitive capacity but low functional capacity, accompanied by high depressive symptoms. We did not observe the opposite pattern, i.e. high functional with low cognitive capacity. These findings align with existing literature, which suggests that intact cognitive function is necessary for functional autonomy (i.e., individuals with severely reduced cognitive capacity will have difficulties performing activities such as using the phone or cooking meals). However, having intact cognitive function does not guarantee functional autonomy: Even centenarians with intact cognitive abilities may exhibit significant functional dependence due to factors such as reduced mobility or other physical health issues [12, 14]. Our results emphasize the importance of identifying subgroups to better comprehend centenarians’ functioning. We discuss the three subgroups identified in the current study in greater detail.

### High-Capacity Profile

The high-capacity profile, consisting of high levels in high functional capacity, high cognitive capacity, and good mental health, which we found in almost half of the sample, aligns with much of the existing literature. For example, studies show a positive association between functional and cognitive capacity in centenarians [11–13] and also that higher functional and cognitive capacities are associated with greater subjective well-being, lower depressive symptoms, and more positive affect [8–10, 33, 34]. Thus, providing additional evidence in line with previous studies [32–35], our findings offer further support to the existence of a subgroup of centenarians who have at the same time high functional and cognitive capacity and good mental health.

### Low-capacity Profile

We also identified a low-capacity profile, with was present in about one third of the sample. Prior research identifying centenarian profiles has also uncovered low-capacity profiles, typically associated with more depressive symptoms. In contrast, our study found that individuals with this profile, despite their poorer functional and cognitive capacity, exhibited the same low levels of depressive symptoms as the high-capacity profile. This may appear surprising, but these results align with literature on psychological adaptation in very old age [23, 49] and previous findings of persistent high subjective well-being despite declines in functional and cognitive abilities [9, 20, 22]. There are multiple aging pathways that can involve compensating for functional and cognitive declines by employing adaptive psychological and social mechanisms. Emotional vitality, resilience, coping strategies, optimism, spirituality, and social connections are all potential compensatory mechanisms that can help an individual sustain high levels of well-being [23, 49, 50].

#### Low-functional-high-cognitive-capacity Profile

The low-functional-high-cognitive-capacity individuals constituted the smallest of the three groups and displayed a distinctive and seldom-explored profile. They demonstrated low functional capacity alongside high cognitive capacity, and elevated depressive symptoms. The finding that nearly all individuals in this group (93.8%) met the criteria for depression, including severe depression (25%), suggests that this profile holds clinical significance. For comparison, a scoping review conducted by Gomes da Rocha et al. (currently under review) computed the prevalence rate of depression in centenarians by aggregating the results of several studies. In a subset of 11 studies that all used the GDS-15 scale and reported depression prevalence in centenarians, the median reported prevalence rate was 27.1%. Unfortunately, most studies do not provide data on the prevalence of severe depression. Given that we identified a group of centenarians in whom we observed a very high prevalence of potential depression compared to what is reported in the literature, it’s worth considering the factors that may contribute to the emergence of this profile.

A possible explanation is that low-functional-high-cognitive-capacity individuals may feel unhappy with their lack of independence due to their high cognitive capacity. This aligns with previous research, which observed that very old adults with higher cognitive functioning and poorer health had a poorer self-perception of aging [51]. In other words, when individuals advanced in age still possess their full cognitive capacity to recognize their deteriorating health, their aging satisfaction was at its lowest. A similar argument could be made for the low-functional-high-cognitive-capacity profile: When centenarians are cognitively well-functioning yet struggle to live independently due to limited functional capacity, their comprehensive grasp of their predicament might undermine their subjective well-being, making them more susceptible to mental health issues such as depression. Alternatively, individuals with high cognitive capacity may also be more adept at reporting depressive symptoms compared to those with lower cognitive capacity. Cognitive decline in the latter group could potentially protect them from experiencing negative emotions associated with their reduced functional capacity or impede their ability to report such feelings. Another compelling reason for depressive symptoms within the low-functional-high-cognitive-capacity group could be high levels of pain and arthritis. In the next paragraph, we elaborate on the potential link between pain, arthritis, functional capacity, and mental health.

### The Impact of Pain and Arthritis

Considering centenarian characteristics associated with the identified profiles, our findings suggest that, while socio-demographic variables such as age, gender, living situation and education played no role, health factors were an important factor in distinguishing the low-functional-high-cognitive-capacity profile from the other profiles. Specifically, these individuals did not report more sensory or mobility issues that could explain lower functional capacity and higher depressive symptoms. However, they reported being affected by arthritis significantly more often (i.e., 9 out of 10 low-functional-high-cognitive-capacity centenarians reported arthritis), and they reported stronger pain. Our analyses indicated that arthritis was associated with diminished functional capacity, in agreement with the literature [37, 40, 52]. Moreover, we showed that when factoring in the effects of arthritis and pain intensity, a more pronounced positive correlation emerges between cognitive and functional capacities than what has been previously documented in the literature [11–13]. This may be attributed to the accounting for cases where individuals, despite having good cognitive capacity, demonstrate diminished functional capacity due to the constraints imposed by arthritis and pain. This sheds new light on the relationship between cognitive and functional capacity, especially in the context of arthritis.

Arthritis, as well as the severity of pain often linked to it, were correlated with increased depressive symptoms. This is consistent with existing studies that have identified both indirect [39] and direct [38] relationships between arthritis and decreased mental health. Thus, our results show that it is important to consider pain when examining the relationships between functional and cognitive capacities, and depressive symptoms. Notably, our findings imply that among centenarians, the role of functional and cognitive capacities in determining mental health is ambiguous, while the presence of arthritis and pain emerge as more prominent determinants. This adds to previous research with the HD100-II sample [28] which reported important pain issues in these German centenarians. Within this sample, 30% of centenarians often experienced pain, and within this group 36% indicated that pain exceeded bearable levels. Because neither functional nor cognitive capacities were still associated with depressive symptoms after adjusting for pain and arthritis, we can surmise that arthritis and pain both directly (i.e., the difficulty of psychologically managing pain) and indirectly (i.e., through reduction of functional capacity) contributed to mental health issues. This finding further extends previous research that reported a weakened relation between functional and cognitive capacities and mental health in centenarians compared to younger older adults [22], and highlights arthritis and pain intensity as potential risk factors for poor mental health outcomes in centenarians.

#### Limitations

The process of conducting face-to-face interviews with centenarians poses significant challenges, including difficulties in recruitment, health complications, cognitive impairments, and fatigue. These difficulties often result in limited sample sizes as observed in our study. Nevertheless, we employed an exploratory analysis method which is viable for small samples, consisting of hierarchical clustering. Our assessment of the clustering outcomes indicated that our method effectively categorized most cases (see Supplemental File 1), though there was some uncertainty for a subset of data points. This highlights the value of our approach in exploring capacity profiles in centenarians, while also emphasizing the necessity for future confirmatory studies to further validate these findings. In addition, because of the challenges of collecting self-report data on depressive symptoms in centenarian participants, the participants that we included in our sample were selective in terms of functional and cognitive capacities. This implies our conclusions may not apply to centenarians with extremely low functional and cognitive abilities. A final limitation to note is the cross-sectional design of our study, which does not establish causality. Any causal relationships should be thoroughly investigated in future research, ideally through longitudinal studies. For example, it is important to better understand the interplay and potentially bidirectional effects between pain conditions and mental health. In addition, it is important to consider depression subtypes, such as recurrent depression or late-life emergence of depression. Even though the data of the current study did not allow to examine depression subtypes, future centenarian research should consider investigating such differences to gain a more complete picture of depressive symptomatology in very old age.

## Conclusions

Our research highlights the importance of identifying profiles or subgroups among centenarians to better capture their heterogeneity in functioning, and comprehend the relationships between functional and cognitive capacities, and mental health. Above and beyond previous studies, our work revealed a low-functional-high-cognitive-capacity group characterized by low functional capacity alongside high cognitive capacity, accompanied by more depressive symptoms. This challenges the idea of simple relationships between these key domains of functioning - physical, cognitive, and mental health - in very old age. Further research is needed to understand why this group experienced more depressive symptoms. Our analysis suggests that neither functional nor cognitive capacity alone were associated with mental health in centenarians, but that pain and pain-evoking conditions such as arthritis have an important role in explaining suboptimal mental health in centenarians. Consequently, identifying and treating pain conditions may enable better mental health in centenarians, but could also help to maintain better daily functioning and cognition, improving aging trajectories for very old individuals.

### Electronic supplementary material

Below is the link to the electronic supplementary material.

Supplementary Material 1

## Data Availability

The data supporting the findings of this study are not openly accessible, in compliance with the data sharing policies agreed upon by the study participants. However, Dr. Daniela Jopp (daniela.jopp@unil.ch) can provide access to the data upon receiving a reasonable request.
