# Supplementary material for: Profiles of centenarians’ functioning: linking functional and cognitive capacity with depressive symptoms
Source: BMC Geriatr. 2024 May 23;24:451. doi: 10.1186/s12877-024-05036-8 (PMC11119272; doi:10.1186/s12877-024-05036-8)
Supplement: Supplementary file 1 — Supplementary Material 1 [file 12877_2024_5036_MOESM1_ESM.docx]

**Supplemental File 1: Evaluation of the cluster solution**

***Ward’s dendrogram and between-clusters inertia gain.***

In our analysis, hierarchical clustering methods were employed to identify groupings within the dataset, utilizing the approach outlined in [1]. The scores for functional and cognitive capacities, along with depressive symptoms, were standardized prior to analysis. Subsequently, hierarchical clustering on principal components was conducted using the HCPC function in the FactoMineR package, using Ward's linkage method coupled with the Euclidean distance measure. The resulting dendrogram, depicted in figure S1, illustrates the hierarchically formed clusters. The height indicates the within-cluster inertia resulting from the merging of clusters. The figure suggests that merging observations into one or two clusters leads to relatively high within-cluster inertia compared to maintaining three clusters. In addition, figure S2 displays the gains in between-cluster inertia when creating additional clusters, revealing a large gain when forming two clusters instead of one, and a similar gain when transitioning from two to three clusters. However, the transition from three to four clusters shows a markedly smaller gain, suggesting that increasing to four clusters does not effectively maximize between-cluster inertia. The level of within-cluster inertia (figure S1) and between-cluster inertia (figure S2) suggest that three clusters represent the optimal clustering solution. Figure S3 visualizes three clusters on the principal component map, highlighting that the first principal component predominantly loads on depressive symptoms (i.e., GDS), and the second principal component on measures of capacity (ADL+IADL and MMSE). Note that in figure S3, S4, S5 and S6, cluster 1 consist of low-capacity individuals, cluster 2 are mixed-capacity individuals, and cluster 3 are high-capacity individuals.


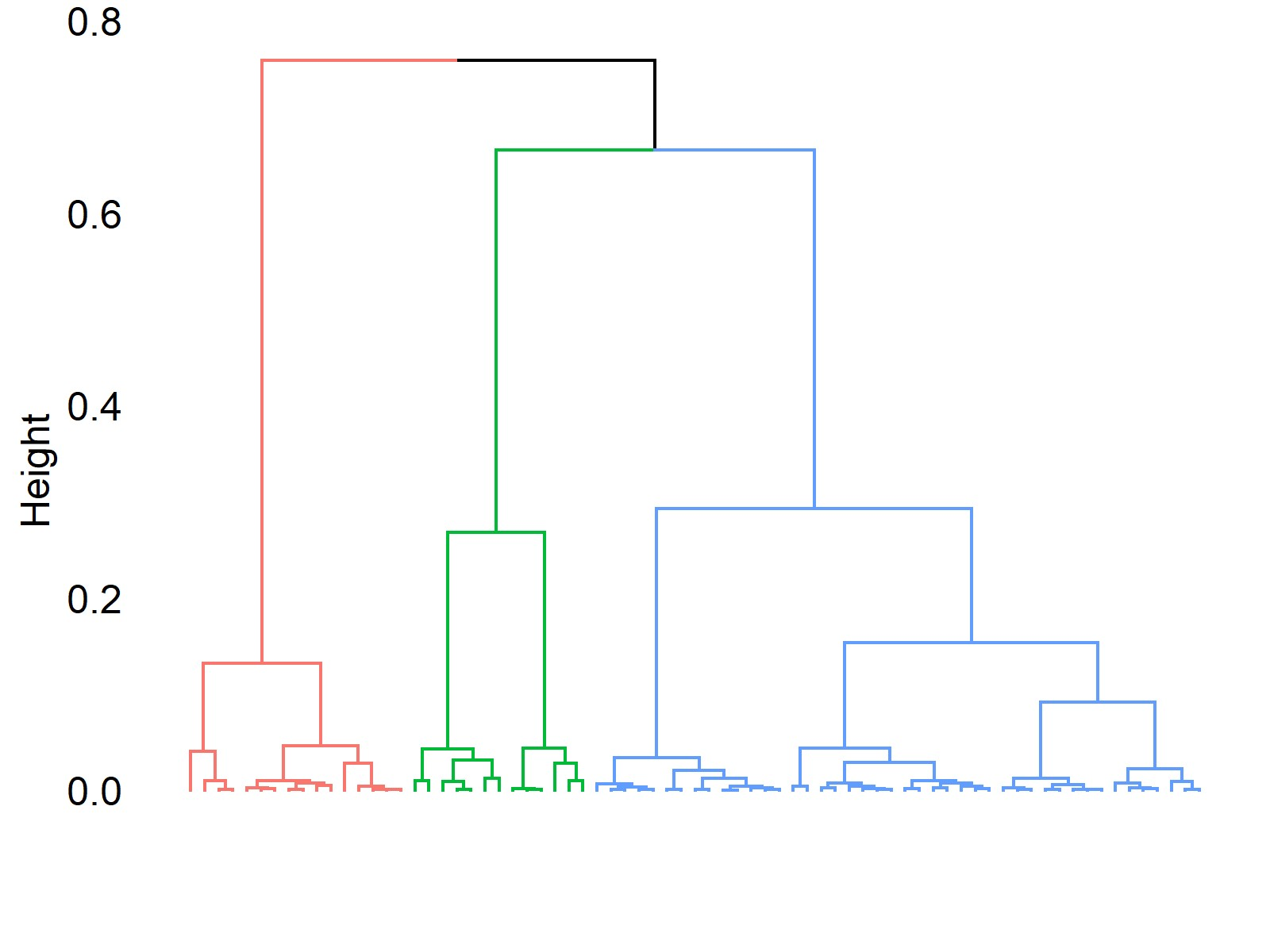


*Figure S1. Ward’s dendrogram. The height represents within-cluster inertia.*


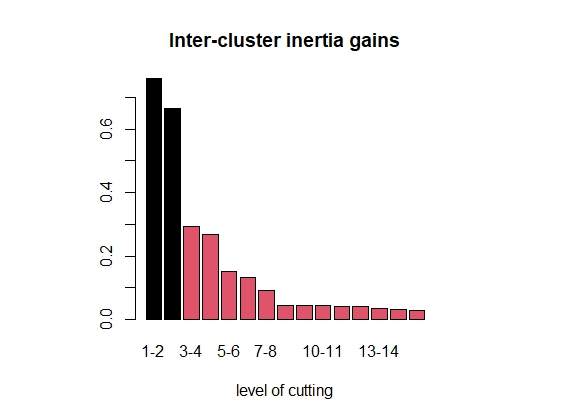


*Figure S2. Between-cluster inertia gains.*


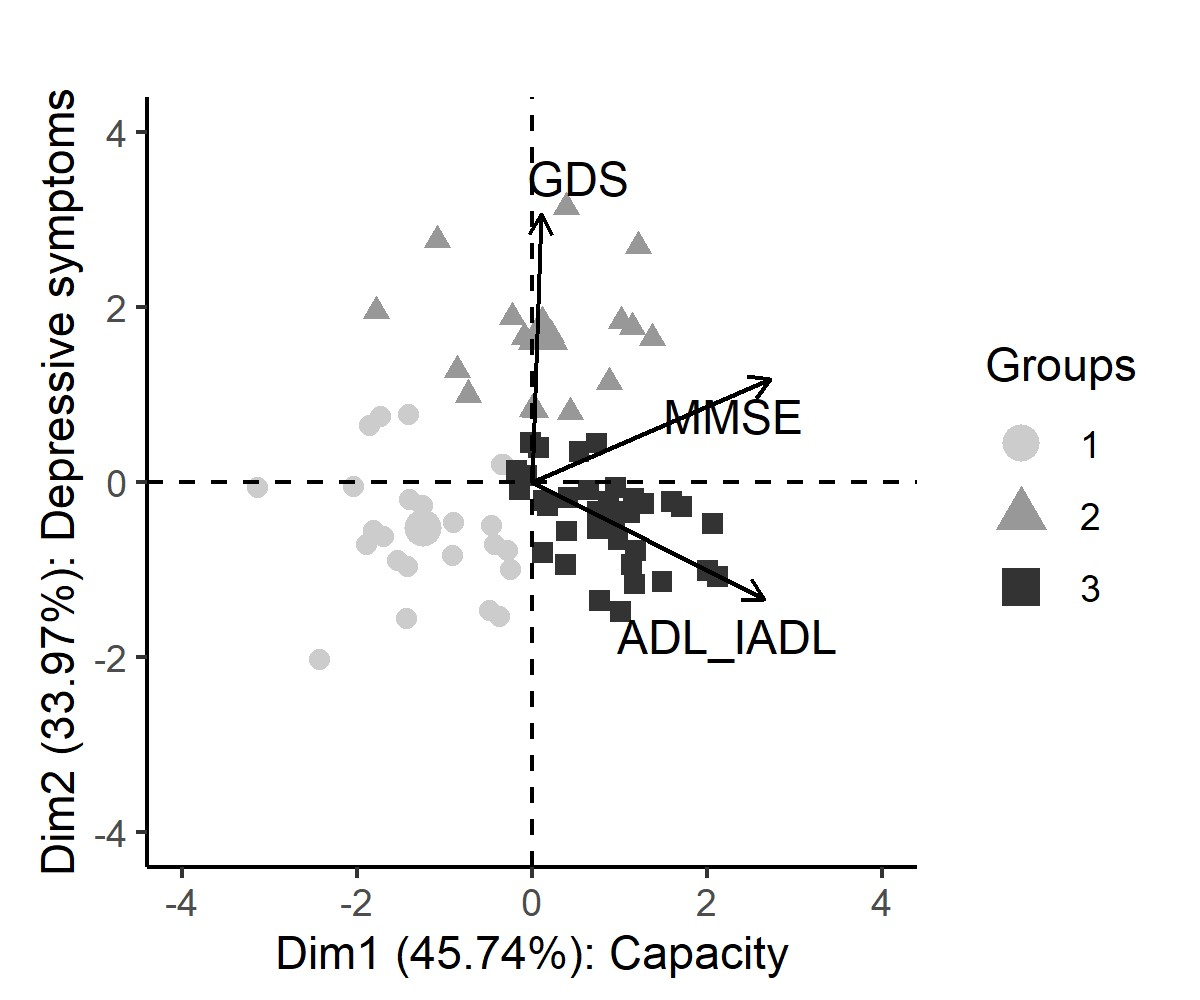


*Figure S3. Principal component map with cluster groups.*

***Evaluation of the cluster solution***

To evaluate the quality of our cluster solution, we conducted silhouette analysis on the three-cluster solution (see figure S4). This involves considering each data point and measuring the average distance to other points in the same cluster (i.e., cohesion) compared to points in the nearest other cluster (i.e., separation). The resulting silhouette coefficient ranges from -1 to 1. Coefficients approaching -1 suggest that points are likely misplaced within their current cluster, values close to 0 indicate points that lie near the boundary between two clusters, and coefficients nearing 1 reflect a clear and appropriate classification of points within their cluster. Our analysis yielded an average silhouette width of 0.34, suggesting a fair clustering structure, superior to solutions with two and four clusters, which had average silhouette widths of 0.30 and 0.29, respectively. This indicates better cohesion and separation for three clusters compared to other solutions. While some observations are near to the boundaries between clusters, indicating more uncertainty in their cluster attribution, most observations fit well within their respective clusters.

To assess the stability of our cluster formation, we implemented a bootstrap method, creating 1000 same-sized samples through random selection with replacement from the original dataset. In each bootstrap sample, certain observations may be repeated once or multiple times, while others may be absent altogether. Each bootstrap sample was subjected to the same three-cluster analysis. As illustrated in figure S5, most observation pairs within the same cluster were grouped together consistently, with an average pairing consistency of approximately 0.80. Conversely, figure S6 indicates that observation pairs from different initial clusters were rarely clustered together in the bootstrap samples, with an average cross-cluster pairing consistency ranging from 0.08 to 0.15. These findings affirm the robustness of our clustering solution, demonstrating its stability across varied sample compositions.


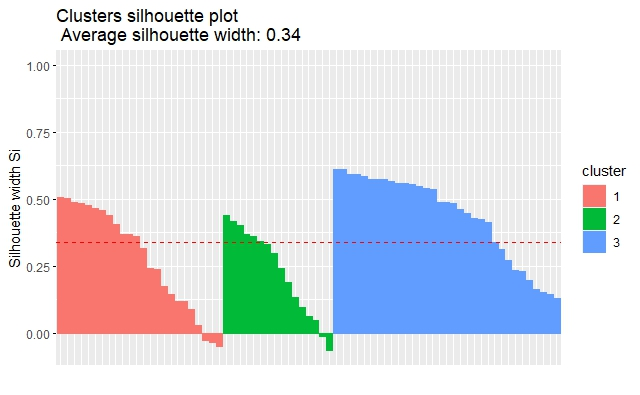


*Figure S4. Silhouette of the three-cluster solution.*


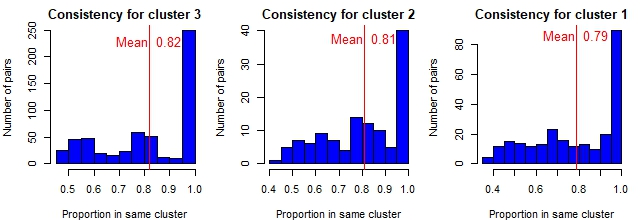


*Figure S5. Consistency of within-cluster attributions.*


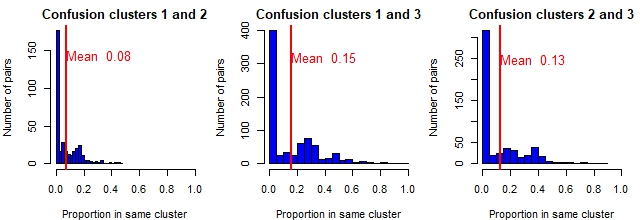


*Figure S6. Between-cluster attributions.*

**References**

1 Husson F, Josse J, Pages J. Principal component methods-hierarchical clustering-partitional clustering: why would we need to choose for visualizing data. Appl Math Dep. 2010;17.
